# Towards work-life balance or away? The impact of work from home factors on work-life balance among software engineers during Covid-19 pandemic

**DOI:** 10.1371/journal.pone.0277931

**Published:** 2022-12-14

**Authors:** Ranitha Weerarathna, Nilmini Rathnayake, Inuri Yasara, Piyumi Jayasekara, Dewni Ruwanpura, Sachindra Nambugoda

**Affiliations:** SLIIT Business School, Sri Lanka Institute of Information Technology, Malabe, Sri Lanka; Asia University, TAIWAN

## Abstract

The paradigm shifts of conventional office spaces for virtual workspaces which practiced Work from Home (WFH) due to Covid-19, created a serious change in the lifestyles of employees, due to the overlap of ‘work’ and ‘life’ domains in one’s life. Since software engineers have a possibility of permanently adapting into WFH, the objective of this study is to unveil factors which would have a significant impact on the work-life balance of software engineers in Sri Lanka, while WFH. Only a very limited researches have shed light on this context, thereby this study would contribute to fill the empirical gap. The study undertook a quantitative approach by collecting primary data through a questionnaire from 384 participants, based on simple random sampling, and analyzing collected data based on Partial Least Squares Structural Equation Modelling (PLS-SEM), using Smart PLS 3.3.9 software. Study results revealed that ‘supervisor’s trust and support’ and the ‘individual workspace,’ have a significant impact on work-life balance, while ‘working conditions,’ ‘possibility to access the organization’s networks’ and ‘number of children’ have no such significant impact. Thereby the study infers that, sound support and trust extended by supervisors and a designated distraction-free workspace; as measures to demarcate the boundary of work and life. Distinctive findings of this study would primarily be fruitful for software engineers to dive into a balanced state of work and life not only during Covid-19 but in future too. Study findings will also contribute to software industry personnel and policymakers in Sri Lanka as well as other developing countries, to establish effective strategies in favor of software engineers who WFH. Further, considering IT industry’s significant contribution towards Sri Lanka’s economic growth amidst Covid-19, results of this study would be high-yielding to indirectly succor IT-services-supported economic growth amidst the pandemic-driven hardships in Sri Lanka.

## 1 Introduction

The World Health Organization (WHO) declared the coronavirus (Covid-19) outbreak a global health emergency on January 30, 2020, and a pandemic on March 11, 2020. The widespread of this pandemic disrupted both economic and social activities of almost all countries. Adhering to health precautions, many workers did not report to work, hence firms’ operations were on halt. With this situation, even in Sri Lanka almost every firm had to shut down either permanently or temporarily [1]. When government-imposed measures to limit hyperinflation, millions of individuals across the globe were compelled to Work from Home (WFH) [2]. As such, the concept of WFH emerged as the best solution to be practiced amidst Covid-19, to support the continuation of business operations, even in the context of Sri Lanka.

Golden [3] defines WFH as a work method that comprises working away from a traditional corporate headquarters for a certain period and using technology as needed to complete tasks. During Covid-19, it was practiced as where the work domain is set out in the context of one’s home itself which ensures the safety of citizens and restricts commuting to control contagion. This concept highlighted a very close connection with the practice of “Work-life balance,” due to the crossover between ‘work’ and ‘life’ domains, whilst WFH. Many past researchers emphasize that telecommuting or WFH had blurred the boundaries between work and life, and employees find it difficult to demarcate the boundaries and times of work and life, when WFH. Past studies elaborate that, the capacity of a specific worker to balance the two domains may vary depending on various factors such as workload factors, family circumstances, conditions, and the worker’s talents [4].

The effect of the relationship between WFH and Work-life balance is much more challenging, from the perspective of software engineers, due to the reason that they can fully attend their job duties, remaining at home, if relevant resources are supplied. Hence, it means that they have the possibility to practice WFH for the foreseeable future. Linking this to the concept of work-life balance, past research studies specify that in a WFH setting, it may be impractical for a software engineer to switch off the laptop at the end of the work shift and transition to personal life in a home-based working arrangement [5], making it harder for them to achieve work-life balance.

Further considering the Information Technology (IT) industry In Sri Lanka, during April–May 2020, its percentage of the establishments of those who were in full operation was at 10.16%, only being second to the agriculture, forestry, and fishing industry. Also, only a minute amount of 20.22% of employees became unemployed during the same period, in the IT industry [6]. This reflects that the IT industry continued operations amidst the Covid-19 pandemic by opting for WFH as soon as the pandemic situation escalated. Currently, almost all the software engineers in Sri Lanka are occupied as teleworkers who WFH. Moreover, considering the continuing pandemic spread and other cost benefits, nearly every IT organization is set to practice full-time WFH for prolonged periods. Thereby, with previous research findings and the prevailing situation in the country, it is quite evident that among the entire workforce, WFH has a major impact on software engineers. Thus, this situation indicates a crucial effect on their work-life balance as well. Considering such importance, it is necessary to examine the factors affecting the work-life balance of software engineers while WFH.

In a study conducted in Sri Lanka examining virtual office platform’s effect on work-life balance, Rathnaweera and Jayathilaka [1] affirm that only limited research publications are available regarding the same variables, concerning the Sri Lankan context. Thereby, as far as we (researchers) are aware, no study has yet been conducted in the above-mentioned sphere of research, on the scope of software engineers, in Sri Lanka. Thus, this study will focus on contributing to the above-mentioned empirical gap.

The sole objective of this study is to unveil which factors significantly affect the work-life balance of software engineers, while WFH. As a result, this study differs from earlier research and contributes to the body of knowledge in four different ways. Firstly, according to researchers’ knowledge, no prior research has been carried out addressing the same in the Sri Lankan context, thereby this study will be the first endeavor on this specific topic.

Secondly, according to the annual report of 2020 published by Central Bank of Sri Lanka (CBSL), IT and telecommunication services sector has played a pivotal role during the year 2020 achieving a growth of 14.1%, amidst Covid-19 under WFH arrangements, hence has performed economically better than other service sectors. Therefore, it is suggested by the CBSL to embrace a permanent adoption of the WFH practice with the aim of achieving better economic growth via increased labor force participation and savings [7]. Thus, findings of this study would be fruitful to step towards enhancing economic growth via IT and related services.

Thirdly, the findings are helpful particularly to software engineers to reap insights on balancing their work and life domains amidst the Covid-19 pandemic as well as any future emergency situations or subsequently as a common practice of work. Software engineers would be in a better position to determine the factors that have a higher impact on their work and life domains. Hence, with much preparedness, they can better manage the effect of those factors, in a more personal and official scope.

Finally, organizations providing software services in Sri Lanka and other developing countries, as well as policy makers of IT industries of those firms, will find the study findings helpful. This will assist them to ensure a satisfied workforce within the companies to achieve company goals, after formulating effective strategies and eventually to have a work-family balanced and satisfied labor force within the countries.

The layout for the rest of the paper would be as follows. Literature review will be addressed in the next section, while section 3 represents materials and methods used in the study. Later section 4 assesses the empirical results with the discussion of results in section 5. Sections 6 and 7 elaborate limitations of the study and concluding remarks, respectively.

## 2 Literature review

In developing the literature review researchers referred to reputed journal databases such as Science Direct, Research Gate, Taylor & Francis online and Wiley online library, etc. Researchers had used WFH, work-life balance, telecommuting, Covid-19, and software engineers as key terms when searching articles. Initially, the literature review consists of the study’s dependent variable; work-life balance. Consequently, it contains the dimensions that the researchers had used to address the concept of WFH; working conditions, supervisor’s trust and support, possibility to access the organization’s networks at home, number of children and individual workspace, which serve as the independent variables of the study.

### 2.1 Work-life balance

A study done in England by Wheatley [8], elaborates that the term "work-life balance" as a person’s capacity to properly juggle job and family duties, irrespective of age and gender. Although work-life balance is defined in many ways, in simple terms, it is about creating a balance between work and life. Another study on 26 software development and IT support teams in companies in different countries starting from Russia to USA, states that the Covid-19 factor drove IT organizations to enable fully remote mode in the spring of 2020. For team leads, project managers, and engineers, traditional communications, task management, and zones of responsibility have been drastically altered by lockdown, self-isolation, and new state regulations in the labor area [9]. With that, balancing the work and life domains had been quite challenging during the pandemic. In a study done in Bangalore, India by Kumari and Devi [10], the term "work-life balance" highlights the differences between the demands of work relationships and home relationships. Another research carried out in the Karnataka state India defines work-life balance as a spectrum in which an individual alternate between both the two extremes of life and work at a point in time [11].

Several factors that affect the work-life balance could be identified. A research study done in the United Kingdom (UK) elaborates on the other factors that affect the work-life balance including the necessity to share electronic gadgets and internet connections with the rest of the family, as well as increased stress levels from more frequent video conferencing [12]. Further, the quarantines and lockdowns affected the relationships between people and resulted in isolation, where low productivity was seen in the work aspect. Research carried out in India by Bhumika [13] confirms that many individuals frequently hire domestic assistants to complete the required household chores, but the lockdown made it tough for the mobility of domestic assistants, which restricted them to perform their duties. In such a situation, the majority of working professionals became overloaded with domestic tasks that ate up their time and energy and probably left them feeling fatigued. This made them not fulfilling their work on deadlines stipulated.

It is demonstrable that with the home converted into a workplace, it has questioned the work-life balance. A Turkey based research points out that, as of now, the home setting is not only for the domain of life but has become a place for work and a school for children. The results pointed out that the WFH concept had led to overworking and an increased number of meetings and reports which raised concerns regarding the physical and mental health of individuals [5]. This had adversely affected the work domain of employees as it diminishes the productivity of work. Further, Rashmi and Kataria [14] prove that with individuals starting WFH, their workload had been doubled, simply because work responsibilities are to be fulfilled amidst household responsibilities. Furthermore, he adds that individuals should pay attention to family responsibilities and ensure that the demands of the household members are fulfilled. With reference to past research done, it can be inferred that in the Sri Lankan context, the work-life balance of software engineers is still under researched.

### 2.2 Working conditions

With the effect of Covid-19, managing all the household work is considered a challenging task while WFH. The difficulty could depend on the working condition of a particular employee who is WFH. Considering these facts researchers have presented various perspectives on working conditions. According to a study based on European Union working conditions, the definition of standard employment has shifted as a result of neoliberal economic globalization [15]. Moreover, a global workforce study (consisting European Union, China, the Republic of Korea, Turkey, the United States, Spanish-speaking Central America, Argentina, Chile and Uruguay) states that due to the effects of globalization, technological advancements, and new organizational strategies for the workplace during the past few decades, the need to monitor and improve working conditions has become more vital. According to Bouncken, Lapidus and Qui [16], a suitable workspace with the necessary infrastructure and technology is needed for the home office. Employee integration with file and task systems, for instance, is possible with groupware (e.g., Slack, Trello, Microsoft Teams). Virtual labor places high demands on IT infrastructure and data security, and it also introduces rigid formal limitations on how work is done. Employees were given the appropriate hardware and software during the pandemic, and accordingly, work processes grew increasingly digital. Apart from this, working conditions must be measured in order to be understand the three basic metrics for evaluating working conditions are compensation, working hours, and contractual agreements [17].

Researchers indicate the positive contribution and the impact of key elements of the working conditions on behalf of the work-life balance. Having favorable working conditions benefits both employees’ well-being and business success which explains that maintaining better working conditions probably positively affects the employees [17]. These will lead to an increase in the quality, performance, and sales of the organization. The study further explains working conditions have an impact on employees’ job satisfaction, productivity, work-life balance, and future actions, including whether or not they decide to remain in their current position. Considering the above statements, it is visible that well-maintained working conditions may positively affect both employee and the organizational perspective.

Several studies have presented the negative effects of working conditions towards work-life balance as well as employee well-being during WFH. An Australian study shows that the flexible labor market has resulted in positions with no restrictions on how long, when, or how quickly people can perform work. As a resource that individuals need for optimal health, the time has been advocated as a social determinant of health. It is because people need time to obtain health services, engage in healthy behaviors, rest, work, and provide for dependents [18]. Research conducted in Japan indicates that working hours (long hours, irregular or shift work, night work, etc.) may cause a work-life imbalance due to insufficient time to maintain a personal life. Poor work-life balance has been proposed as a mediator between working hours and health-related outcomes. Lack of time is linked to harmful habits such as poor diets, excessive alcohol consumption, smoking, and lack of exercise [19]. Furthermore, not having enough time to recover from work exhaustion might lead to poor mental health and sleeping issues [20]. Low wages, as a result of fewer working hours and temporary jobs, too can lead to financial insecurity, which can affect one’s health [21]. Therefore, satisfaction with work-life balance is a well-being indicator of public interest [20].

The studies conducted in various countries demonstrate different perspectives and the aspects that have positively and negatively affected the working conditions directly impacting work-life balance during WFH. Hence, considering the above findings of the literature, we propose the first hypothesis of the study:

**H**_**1**_. There is a significant impact of working conditions on work-life balance of software engineers.

### 2.3 Supervisor’s trust and support

The Covid-19 pandemic made almost every individual shift from their usual workplace to their home, which is referred to as the WFH. Since employees continue doing their work at home, the extent of employee–supervisor interaction and support can vary and fluctuate. The amount of support that subordinates receive from their employer is measured by supervisor’s support [22]. Supervisory support is a crucial element in organizational culture. Managerial attitudes and practices are both reflected and shaped by organizational cultures [23]. A study conducted by using the data collected from correctional officers in Taiwan further explains that employees outlook on the quality of their relationship with their supervisors is reflected in supervisor’s support. This indicates how much they feel their superiors are concerned about their personal needs and well-being [24]. A study conducted in UK and Netherland indicates that supervisors can encourage or hinder employees’ capacity to exercise rights and utilize opportunities for a better work-life balance [25].

Further studies emphasize the importance and the positive effects of supervisor’s support on behalf of employees when they are struggling with office responsibilities and work-life balance situations. According to Skiba, and Wildman [26], supervisor’s support is critical in assisting employees in dealing effectively with ambiguity at work. It means employees can rely on their supervisor to help them deal with the uncertainties that make them feel more confident. Research by Abendroth and Dulk [27], proves that when employees feel comfortable talking with their supervisors about work-life balance situations, supervisors provide emotional support when they show empathy for their employees. Supervisors who mentor employees by sharing ideas or counseling them on how to balance work and family life, for example, may leave work at six o’clock to have dinner with his or her family. According to Bailyn [28], work can be rearranged to promote both workers’ work-life balance and the organization’s effectiveness.

Taking into account the above-mentioned statements, it is visible that supervisor’s trust and support for their employees to maintain work-life balance is vital. Thereby, referring to the above-mentioned findings of the literature, researchers commonly assume that the supervisor’s trust and support has a significant impact on work-life balance. Apart from this, as per researchers’ perspective, supervisor’s assistance is particularly vital in maintaining employees’ work morale and psychological well-being during a crisis. Relating these pieces of literature to the current study, we postulate the following hypothesis:

**H**_**2**_. There is a significant impact of the supervisor’s trust and support on work-life balance of software engineers.

### 2.4 Possibility to access organization’s networks at home

Some enormously successful outsourcing and staffing firms have been offering solely remote software development services, creating these services in the best possible ways since 2015 [9]. Also, during the Covid-19 outbreak due to advances in information and communication technologies (ICT) many workers were able to WFH. In this setting, ICT provides them with the knowledge and tools they need to perform duties, finish projects and collaborate with coworkers or business partners outside of their home office. Golden [3] analyzed previous research to uncover the work-home conflict that teleworkers face and offered industry norms for remote workers who successfully deal with balancing work and life. However, adapting to the home-based work environment may require attention to data privacy and protection, a likely challenge for every data-centric organization. Having said that, the study conducted by Golden [3] hasn’t focused on the technology adaptation hardships and limitations an organization may face. In an era where most businesses practice WFH and rely on online platforms, risks arising from data loss and threats to data privacy can outweigh the benefits gained via ICT. Specifically, and unknowingly, this issue can add to the existing burden of employees when WFH.

To carry out day-to-day operations, software engineers require office equipment, adequate workspace, and internet access. But according to Anderson and Kelliher [12], family members may be required to share computer equipment (laptops, tablets, and printers), internet access, and work and study space at a desk or table. This means that a few devices are insufficient to be shared among many family members when WFH. Moreover, as practical implications for future research, these scholars stressed the importance of recognizing the additional stressors and difficulties of attempting to WFH during a pandemic, in addition to the existing difficulties of locating a suitable workspace, accessing equipment, and the dependability of internet connectivity.

However, due to the pandemic, even those who did not have work infrastructure at home were forced to WFH with extremely limited resources. From the researchers’ point of view, software engineers found this situation much harder than the other sectors, as physical infrastructure and internet connections are crucial resources and interrelated to almost every task included in their job description. Based on his research carried out in India, the author has demonstrated how employees faced hardships due to poor network connections and a lack of physical infrastructure, which led to problems in accessing organization networks. Overall, the challenges faced are interlinked as well as the negative outcomes [29].

Aside from the lack of facilities provided, some researchers have highlighted that the lack of authority is interrelated with difficulties in accessing an organization’s network at home. According to Sellar and Peiris [30], many studies have identified the lack of authority and accessibility of an organization’s network as a disadvantage, and all relevant documents should be available online to produce the intended outcomes from work done by employees who WFH. Furthermore, providing employees with suitable technology and tools has a favorable impact on the success of telework [31]. Although some employers have offered accessibility and autonomy to employees, productivity may decrease due to a lack of experience handling networks without supervision. Even with the necessary infrastructure in place, some researchers claim that software engineers have trouble adjusting to the remote working environment. Therefore, it is possible that companies that reflect the ideal employee situation in this pandemic and meet opposing objectives are more likely to succeed.

**H**_**3**_. Possibility to access the organization’s network at home have a significant impact on the work-life balance of software engineers.

### 2.5 Number of children

Unlike other variables considered for this study, the number of children is a gender-sensitive variable, affecting female employees with children more than male employees. It is assumable that WFH might be a tough task when there are children at home since they should be given attention while fulfilling employees’ work and life responsibilities. Moreover, children too are affected by physical classrooms replaced by online education, where much burden including supervision is piled upon working mothers. In a German study [32], stated that concerning the WFH behavior and performance of employees, their children play a vital role. This statement was further supported by several pieces of literature, by citing that the number of children is a fundamental factor to be considered in the context of WFH in countries such as Sri Lanka, the USA, and Germany [1, 33, 34]. However, the study conducted by Rathnaweera and Jayathilaka in the context of Sri Lanka has only focused on linear impacts, where non-linear relationships were not taken into account. It was proven by much literature that women who are married and have children show a higher tendency to WFH, when compared to married women who do not have children, for the sake of balancing their work and life [1, 32].

Various literature expresses the fact that having children at home would worsen the work-life balance. In a study conducted in USA, Germany and other 38 countries, it has proved that mothers who have children of less than 18 years, experience a higher degree of conflict between work and life [33]. In a Moroccan study Semlali and Hassi [35] concludes that, since females give a higher priority for family-related matters, when disputes arise between work and family, females who have children are highly affected by the conflicts that arise between work and life. This would eventually hinder the work-life balance of female employees married and with children.

Simultaneously, while several pieces of literature state that having children diminish work-life balance, Crosbie and Moore [36] lay out a better aspect of work-life balance. Here, they state that employees who have children and are WFH have had the opportunity of developing better and closer relationships with their children and were capable of taking care of them as well. At the same time, a Romanian study has proven that there is no significant effect on work-life balance by the presence of children [37]. Through the findings of the above literature, the impact of the number of children on work-life balance gives an inconsistent impression. With mixed findings in this regard, considering Sri Lanka-related findings from the above literature, researchers commonly suggest that the presence of children affects work-life balance which has not been tested so far in the context of software engineers. Thereby, the researchers propose the following hypothesis to be tested in the Sri Lankan context in the perspective of software engineers.

**H**_**4**_. Number of children has a significant impact on the work-life balance of software engineers.

### 2.6 Individual workspace

With the pandemic affecting globally, individuals had to shift their workspaces to their homes. According to a Turkey based study, Tokdemir [5] affirms that the change of the workspace too will affect the balance between work and life domains. Simply, this is because both the domains are under operation in the home environment, where it is now a workplace, a school, as well the space for the entire family and their activities. This shift of individual workspaces from a corporate office to the home office had affected individuals differently: where WFH had been a better option for some and the opposite for others. Anderson and Kelliher [12] in a study done in the US, describe that WFH or remote working is considered to be relaxing and rather productive. This is because it lends ample time to take breaks between work and focus on work peacefully. Researchers point out that with the time flexibility in WFH, employees don’t have a set of proper working hours unlike in a physical office space. This situation may see hours spent on work and personal life of employees overlap, thus, the need for individual workspace is becoming increasingly critical.

Although some people manage to perform well in both contexts when at home as well as the workplace, some find it difficult to do so. Shirmohammadi, Au and Beigi [38] infer in a study done in various countries like Australia, China, and others, that despite individuals having the opportunity and flexibility to work at home rather than returning to their traditional workplaces, not every individual has the privilege of having a designated home office or even an individual workspace. Therefore, employees have no option other than managing work and family and also facilitating the space at home among family members to meet their demands. Thereby, individual workspace could be considered as a requisite for better work-life balance and to uplift productivity while telecommuting. Based on a US study, Golden [3] stresses that to maintain a work focus and avoid household routines by family members (such as cooking and cleaning) posing as interruptions, it is necessary to have a separate area for work. But not everyone is capable to afford or to have space, especially for those who live in apartments. A lack of work focus can result in poor performance of employees, even those who perform well in a physical office space.

Researchers point out that the working environment highly impacts the success or failure of meeting deadlines and fulfilling the family responsibilities of employees. According to previous literature, individual workspace had impacted the balance between work and life. As such, after much consideration, the following hypothesis is put forward to be tested in the Sri Lankan context from the perspective of software engineers.

**H**_**5**_: Individual workspace has a significant impact on the work-life balance of software engineers.

The S1 Appendix is based on the above-described key factors of WFH in order to highlight the gaps that the researchers have addressed through this study.

**Fig 1:**
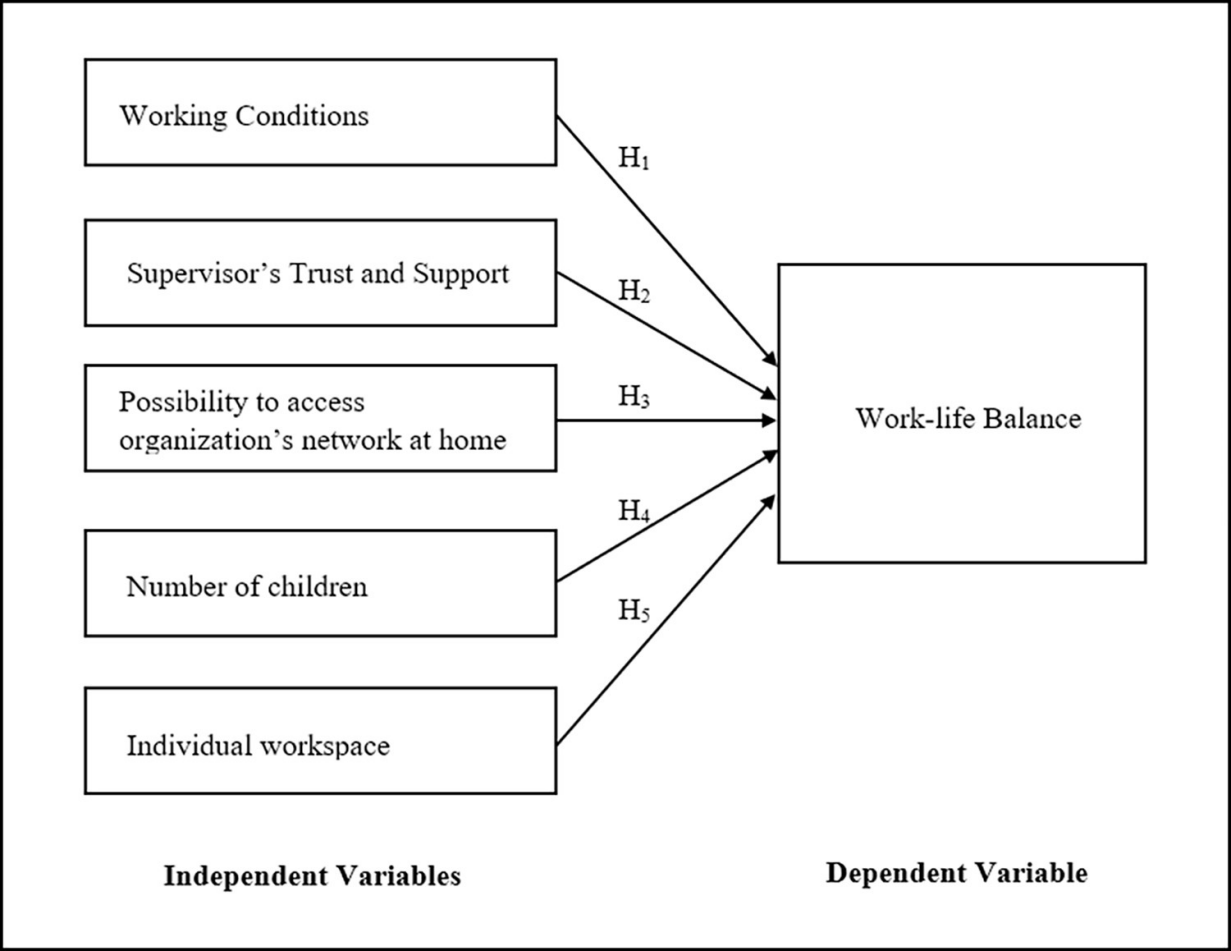
Conceptual framework.

Based on the above-developed hypothesis, researchers developed the following conceptual framework for the study (Fig 1).

## 3. Data and methodology

The SLIIT Business School examined and approved this study. Later, the study was carried out following a quantitative approach-based framework to analyze data. The entire study design process including data collection method and methodology can be referred through the S2 Appendix.

Data were collected primarily through means of a questionnaire to operationalize the latent variables used in the study. Participants in the experiment granted their verbal consent prior to completing the online questionnaire and were allowed the option to withdraw from the study at any time before their data were anonymized. No incentives were provided for any of the respondents to take part in this study. In a similar vein, all authors and participants in this study stated that they have no conflicts of interest related to the investigation.

The population for the study consists of the software engineers who WFH in companies providing software development services, registered under the Sri Lanka Export Development Board (EDB) [39] and operating in the Western Province of Sri Lanka. As per EDB, currently, 167 software development companies are registered in Sri Lanka. Yet only 119 software development companies are established in Western Province, which currently operates and were reachable. Within the said 119 software development companies, approximately 9,402 software engineers are employed, which form the study’s population. Hence, the sample size for the analysis was taken as 384, in accordance with the Table expressed by Krejcie and Morgan [40].

The participation of respondents in the study was completely voluntary. Thereby, researchers adopted a simple random sampling technique to avoid any biases when collecting data. A pilot survey was carried out in April 2022, using 43 respondents to confirm the clarity, understandability, and logical order of the questions. Consequently, the survey was carried out later in May 2022 by sending out 384 questionnaires for randomly chosen software engineers employed under the selected 119 EDB registered software development firms in the Western Province of Sri Lanka. Thereby, the unit of analysis for the study is the software engineers employed in software development firms in the Western Province of Sri Lanka. To collect data, the link of the questionnaire was distributed through the means of emails and prominent social media from April-May 2022. With a response rate of 81.77%, 314 responses were received and included in the analysis of the study. The S2 Appendix can be referred to understand the elimination and inclusion of respondents of the study.

Data collected through the questionnaire survey were analyzed using Partial Least Squares Structural Equation Modelling (PLS-SEM) technique to generate the results on the five hypotheses of the study (S2 Appendix). PLS-SEM is a popular technique in various fields as a key technique for the multivariate analysis approach [41]. This method is crucial to analyzing latent variables’ causal effects in similar past literature by adapting alike approaches and techniques to analyzing such latent variables [42–45]. Smart PLS 3.3.9 software is used as the analyzing tool in this study. As per PLS-SEM methodology, the analysis is carried out in two main steps. First, the measurement model will be validated and then the structural model [44].

## 4. Results

### 4.1 Descriptive findings

The descriptive statistics for the dependent and independent variables of the total 314 participants are depicted in Table 1. Considering the first indicator of WFH which is working conditions, on average the item code (WC1) has the score of (3.828) and a standard deviation of (1.095) which is significantly higher compared to other item codes. The next indicator, which is supervisor’s trust and support, on average item code (STS2) is (3.908) which is higher than others but interestingly item code (STS3) has the highest standard deviation of (0.98). Possibility to access organization networks which is the third indicator, on average item code (PAON3) has a high score (4.137) when compared to other indicators. When considering the standard deviation item code (PAON3) has the topmost score which is (0.967). When moving on to the next indicator, which is number of children, item code (NOC1) has the highest mean score (3.57) and the standard deviation (0.982) in comparison to the other item code. The final indicator of WFH which is individual workspace shows that on average the item code (IW2) is (3.933) but it is seen that the item code (IW1) has the highest standard deviation (0.967). Taking into account the dependent variable which is work-life balance the item code (WLB7) has the highest mean score which is (3.5) and item code (WLB5) has the highest standard deviation (3.934). After considering the average mean score of each indicator of the independent variables the item code (PAON3) affects the WFH the most. Which falls under the possibility to access organizations networks. The least impact is created by the Item code (NOC2). In view of standard deviation, the item code (WC1) affects the most in comparison to others and the item code (PAON1) affects the least. In conclusion it can be inferred that both the mean score as well as the standard deviation depicts the significance of the indicators of the variables.

**Table 1:** Descriptive statistics of indicators’ items.

	Mean	Median	Min	Max	Standard Deviation
WC1	3.828	4	1	5	1.095
WC2	3.640	4	1	5	1.092
WC3	3.691	4	1	5	0.989
STS1	3.860	4	1	5	0.913
STS2	3.908	4	1	5	0.917
STS3	3.745	4	1	5	0.980
PAON1	3.949	4	1	5	0.873
PAON2	3.825	4	1	5	0.967
PAON3	4.137	4	1	5	0.905
NOC1	3.570	4	1	5	0.982
NOC2	3.675	4	1	5	0.956
IW1	3.634	4	1	5	1.048
IW2	3.933	4	1	5	0.967
WLB1	3.076	3	1	5	0.885
WLB2	3.369	3	1	5	0.873
WLB3	3.475	4	1	5	0.907
WLB4	3.424	3	1	5	0.815
WLB5	3.140	3	1	5	0.934
WLB6	2.920	3	1	5	0.919
WLB7	3.500	4	1	5	0.932

Source: Authors’ compilation based on Smart PLS output.

### 4.2 Measurement model results

Researchers validated the outer model by examining the construct’’ reliability, convergence, and discriminatory validity.

#### 4.2.1 Reliability statistics

The Cronbach’s alpha value is used to determine the survey’s reliability; when the Cronbach’s alpha is more than 0.7, the survey is considered reliable. The Smart PLS program was utilized by the researchers to measure the reliability. According to this study’s findings, the Cronbach’s Alpha of working conditions variable is 0.80 which is greater than the threshold of 0.7, therefore, is considered to be reliable. The supervisor’s trust and support variable is 0.90 which is also greater than 0.7, reconfirming reliability. The Cronbach’s alpha is 0.83 which is greater than 0.7 of possibility to access organization network and it is considered reliable. According to Hinton [46], the alpha score being in the range of 0.50 to 0.75 is considered as moderately reliable. The number of children variable is 0.55 and individual workspace variable is 0.50 which is in the range of 0.50 to 0.75, therefore, are considered reliable. The work-life balance variable’s Cronbach’s Alpha is 0.86, which is higher than the 0.5 criterion. Since all of the variables are higher than 0.50, we can conclude that these variables are reliable and hence have internal consistency among the items employed. The Cronbach’s Alpha of the variables is shown in the Table 2.

**Table 2:** Reliability statistics of latent variables.

Latent Variable	Cronbach’s Alpha
Working Conditions	0.80
Supervisor’s Trust and Support	0.90
Possibility to Access Organization’s Network at home	0.83
Number of Children	0.55
Individual Workspace	0.50
Work-life Balance	0.86

Source: Authors’ compilation based on Smart PLS output.

#### 4.2.2 AVE statistics

The validity of an instrument is assessed using the SEM technique in Table 3 employing two basic tests: convergent validity and discriminating validity. The result of the Average Variance Extracted (AVE) scores is used to examine and measure the survey’s convergent validity. Only when AVE scores are larger than 0.5, the validity range is acceptable. The smart PLS program gave scores of working conditions variable as 0.64, supervisor’s trust and support variable as 0.83, possibility to access organization network variable as 0.75, number of children variable as 0.66, individual workspace variable as 0.67 and work-life balance a score of 0.54. Since the AVE score for all variables are greater than 0.5, it is visible that the convergence validity of results are ensured.

**Table 3:** AVE scores of latent variables.

Latent Variable	Average Variance Extracted (AVE)
Working Conditions	0.64
Supervisor’s Trust and Support	0.83
Possibility to Access Organization’s Network at home	0.75
Number of Children	0.66
Individual Workspace	0.67
Work-life balance	0.54

Source: Authors’ compilation based on Smart PLS output.

#### 4.2.3 Discriminatory validity

According to Sekaran [47], discriminatory validity determines whether the constructs in question are genuinely distinct from one another due to reduced correlations, confirming that the two constructs should be examined independently. Either one of three aspects could be used to analyze the discriminatory validity, namely, Fornell-Larcker Criterion, cross loadings or Heterotrait-Monotrait (HTMT) ratio. Researchers have used the last technique of evaluating discriminatory validity, which is the HTMT ratio in determining the discriminatory validity. According to Table 4, working conditions and supervisor’s trust and support, possibility to access organization network, number of children, individual workspace and work-life balance was calculated and found to be lower than 0.9 confirming to be discriminatory valid. Further, the HTMT ratios between supervisor’s trust and support and possibility to access organization network, number of children, individual workspace and work-life balance were lower than the threshold of 0.9 proving discriminatory validity. Also, the HTMT ratios of possibility to access organization network and number of children, individual workspace and work-life balance confirms to have discriminatory validity since these are less than 0.9. Moreover, the HTMT ratios between number of children and individual workspace, work-life balance were lower than the threshold of 0.9 and possess discriminatory validity. Finally, the HTMT ratio between individual workspace and work-life balance is 0.555 which is lower than 0.9 threshold. Therefore, the results point outs that all the constructs possess discriminatory validity.

**Table 4:** HTMT ratio of latent variables.

	WC	STS	PAON	NOC	IW	WLB
WC						
STS	0.228					
PAON	0.354	0.839				
NOC	0.641	0.344	0.492			
IW	0.244	0.511	0.557	0.767		
WLB	0.103	0.495	0.404	0.242	0.555	

Source: Authors’ compilation based on Smart PLS output.

### 4.3 Structural model results

The structural model of the survey was tested by the researchers through the bootstrapping technique in order to identify the significance of the relationship between the latent variables. The beta value, t-statistic, and the p-value of the relationship between the five above-mentioned variables on work-life balance were calculated using bootstrapping with 500 sub-samples and a significance level of 0.05 and presented in Table 5.

**Table 5:** Path coefficients of the relationships.

Relationship	Beta Value	T-Statistic	P-Value
Working conditions -> Work-life balance	-0.009	0.094	0.925
Supervisor’s trust & support -> Work-life balance	0.349	4.702	0.000
Possibility to access organization’s network at home -> Work-life balance	0.009	0.109	0.913
Number of children -> Work-life balance	0.002	0.032	0.974
Individual workspace -> Work-life balance	0.256	4.102	0.000

Source: Authors’ compilation based on Smart PLS output.

The significance of the relationships could be determined if the beta value is greater than 0.20, the t-statistic is greater than 1.96 and also if the p-value is less than 0.05. The Table 5 depicts that the beta value which is-0.009, t-statistic which is 0.094 as well as the p-value which is 0.925 confirms that the relationship between working conditions and work-life balance is insignificant as well that working conditions has a negative impact on work-life balance. Further, it is verified that the relationship between supervisor’s trust and support and Work-life balance is significant. Further it confirms that supervisor’s trust and support have a positive impact on work-life balance as the beta value is 0.349, the t-statistic is 4.702 and the p-value is 0.000. When considering the relationship between the possibility to access organization’s network at home and work-life balance the results confirms that the relationship is inferred as insignificant as the beta value, t-statistic and p-value are namely, 0.009, 0.109, and 0.913. With the beta value being 0.002, the t-statistic being 0.032 and the p-value being 0.974 it can be confirmed that the number of children does not have a significant impact on work-life balance. Finally, when examining the relationship between individual workspace and work-life balance the beta value which is 0.256, the t-statistic which is 4.102 and p-value which is 0.000 shows a significant interrelation.

**Fig 2:**
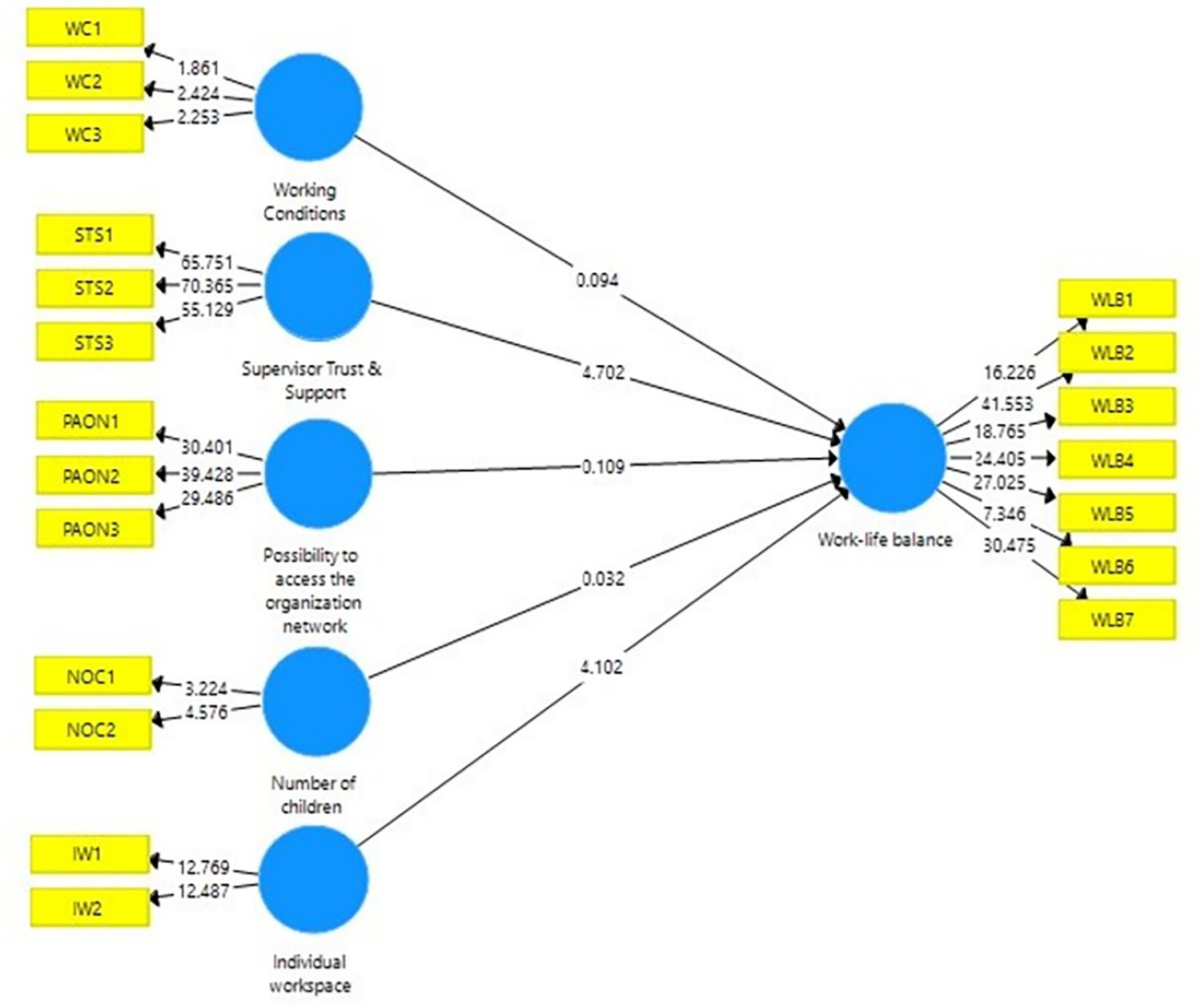
PLS results of the structural model.

Taking into account above interpretations based one the survey results in the Table 5 it is possible to conclude that, from all the above five variables, only supervisor’s trust and support and the individual workspace have a significant relationship on work-life balance, whereas working conditions, the possibility to access organization’s network at home, and the number of children have an insignificant relationship on work-life balance. The structural model of the analysis can be depicted in Fig 2.

## 5. Discussion

The study was conducted to identify which factors significantly impact on the work-life balance while working from home, in the perspective of software engineers, a group of employees who are fully capable of working virtually with the availability of all facilities. While addressing the concept of WFH using five different factors, the study’s results demonstrate that supervisor’s trust and support and the individual workspace that an employee possess, have a significant impact on the work-life balance of software engineers, while remaining factors; working conditions, possibility to access organization’s networks and number of children do not have a significant impact on the same.

The results express that out of the factors used to address WFH, supervisor’s trust and support and individual workspace have a significant impact on the balance between work and life of software engineers. When considering supervisor’s trust and support with a p-value of 0.000, the results confirm the claims of Abendroth and Dulk [27], that supervisor’s support does impact on work-life balance based on a study carried out in the European context. This finding is further supported by a study in a Slovenian hospital [25] concluding that supervisory support helps to achieve an increment in work-life balance. Focusing the current study, software engineers’ job duties are concentrated to be carried out as teams, where team leads are acting out as supervisors. Therefore, the support and trust extended by supervisors for software engineers could be a vital factor for them to perform better and hence to achieve a better balance between work and life. As such, by considering the results, the expectations of the researchers have been fulfilled regarding this factor. Rathnaweera and Jayathilaka [1] in their study which was conducted in Sri Lanka, have considered individual workspace to be measured as a non-working environment factor, and found out that it does significantly impact on the work-life balance, where this study’s result is in line with. Golden [3] in his study too points out that to have a separated dedicated workplace within the home as a best practice when WFH.

Results showing that working conditions do not have a significant impact on work-life balance is surprising, compared to previous studies carried out. Since Wolor, Nurkhin and Citriadin [48] have identified in a study in Indonesian context that having the right conditions while WFH does have a significant impact on work-life balance. A study conducted in Japan by Bannai and Tamakoshi [19] claim that higher working hours would result in an imbalance of work and life. The result for this hypothesis was unexpected by the researchers. Further the study results that possibility to access organizations networks also does not have a significant impact on work-life balance. To our knowledge only a very few studies have looked into the relationship between these two variables, yet the same construct was investigated by Sellar and Peiris [30] and identified that possibility to access organizations networks does not significantly affect to job satisfaction, which could be viewed as one aspect needed to balance work and life. Focusing on the remaining construct: number of children, it has resulted in this study to have no significant impact on work-life balance. This result agrees with the findings of Panisoara and Serban [37] in a study conducted in Romania, that the presence of children has no significant impact on the work-life balance. Yet the result contradicts the findings of a Sri Lankan study conducted by Rathnaweera and Jayathilaka [1] claiming the contrary. The divergence of this result might be due to the differences in context.

Since this study particularly focuses on software engineers who are working from home, who’s job tasks are structured in a way that it should be performed in teams, the support extended by supervisors in their teams have been found significant. Den Dulk and others [25] explain that, the capacity of utilization of opportunities to achieve a better work-life balance can be altered by the supervisors. Building upon this finding, supervisors in the IT industry in Sri Lanka, have a critical role to play when their subordinates WFH. The workspace that an employee has to carry out job tasks was found to be another crucial factor to be considered. According to Golden [3] it should be a separate space as the physical workspace boundary that has a door which can be shut. Studies express that not all WFH employees have the privilege of enjoying such a separate workspace [38]. However, this study’s results emphasize that such designated workspace is of vital need to achieve work-life balance. Especially in the context of software engineers, having an individual workspace with closed doors and free of distractions is vital for them to have efficient collaboration with co-workers. Having a separate space would also help employees to draw an imaginary line between the family and work, so as to focus fully on work while within the workspace, and to give focus on family when otherwise.

The results of the impact of working conditions on work-life balance does not fit with the previous study results. A study conducted on IT professionals in a large Asian IT company found out that working hours and time spent on meetings have been increased during WFH, hence has resulted in a decrease of productivity, and is anticipated that this might impact on work-life balance [49]. Bannai and Tamakoshi [19] claim there is a significant impact too. However, the contrary of the results might be due to the differences in the context. Moreover, it should be stated that most firms considered in this study jointly operate with foreign firms, where Sri Lanka is the regional hub or a development center. Here, parent companies demand for high service quality, therefore software engineers already have long working hours. Therefore, this aggravates the existing issues on work-life balance. However, since this study was carried out in the mid of the pandemic hit, not in the very beginning, maybe the effect of change of working conditions was not that significant for the participants in this study. Islam [29] argues that it is required to have proper equipment and connectivity when employees WFH. Sellar and Peiris [30] claim that all required documents should be available online for the employees to carry out their job tasks while working from home. However, the study’s results infer the contrary. It might be due to the fact that even though it is a vital factor to be present considering the WFH concept, it might not have a significant impact on the concept of work-life balance, as resulted in this study. However, this might be an area where future researchers can shed light into. One of the most unexpected results considering the Sri Lankan context was to have no significant impact of number of children on work-life balance. Many researchers [1, 33] have pointed out that having children significantly impacts on work-life balance. In Sri Lanka, culturally it is a practice that females bear a more portion of family and child responsibilities. And since this effect would be greater on WFH mothers, researchers anticipated that the result of this relationship would be significant. However, the results proved the opposite and hence, Panisoara and Serban [37] study’s results were re-assured from this study.

Based on current study’s results, software engineers who WFH should consider crucially about the support and trust that they have receive from supervisors and individual workspace, since it creates a significant impact on balancing their work and life. Considering this aspect, more trust-extending and support providing superiors should be molded by organizations through policies and practices, supervisors should be trained to work closely and assist well for subordinates, which would ultimately have an effect of creating a well work-life balanced workforce. Further, the workspace that an employee has should be of a well-separated, distraction-free area where the employee could work with 100% focus on work to fulfill job duties as tasks since this also significantly impacts on work-life balance. Moreover, it can be also considered as a partial responsibility of other members within the house premises to provide a distraction-free environment for the employee to work calmly. Other than the current study, certain other studies have adopted the qualitative approach in addressing the same research concern. According to a study by Butler and Jaffe [50] done in the USA, a lesser possibility of being content is one of the many challenges, in addition to one’s mental and physical health issues, work-life balance, and other factors. Further, a study 53points out that, some developers claim to be more productive while WFH, while others claim to be less productive, even if overall reported productivity has not changed significantly [51]. The key factors highlighted in the qualitative studies of WFH during Covid-19 are mental and physical health and employee productivity. These results should be taken into account when formulating policies by the organizations, in order to extend better support by supervisors for WFH subordinates. Further, not merely software engineers, other employees employed in IT related companies, or any other WFH employees could reap out of the results of this study to have a better balance between work and life. The S3 Appendix includes the summaries of all the literature used to justify this study in accordance with its objective.

## 6. Conclusion

As the WFH concept becomes increasingly central to software engineers’ daily lives due to the pandemic, it is important to understand how it affects them when balancing their family life in a home-based work environment. Software engineers fall under software development and the IT sector also have all the necessary resources to carry out their work tasks from home without any interruptions. This study was carried out with the objective of unveiling the impact of WFH factors on work-life balance by testing the working conditions, supervisor’s trust and support, ability to access the organization’s networks at home, number of children, and individual workspace variables, considering them as the factors that most affect WFH. The study resulted that supervisor’s trust and support and individual workspace significantly affects the work-life balance of software engineers. Therefore, software engineers in Sri Lanka, software services provider organizations in Sri Lanka, employees WFH who belong to other industries in Sri Lanka, future researchers and community could gain insights from this study, on the variables that affect the most to WFH. In doing so, these parties would be able to have a better balance between work and life.

The current study highlights the importance of concentrating on factors included both in the working environment and the family environment as key success factors. The findings support some of the past studies and deviate from some others as this study was carried out considering an untouched area of software engineers in the Sri Lankan context. Since the supervisor’s trust and support factor has a p-value of 0.000 which is less than the significance level of 0.05 it turned out to be a significant variable, findings suggest that software engineers have to focus more on their supervisor’s trust and support to maintain a proper work-life balance. By enabling the WFH concept, a variation is visible in the interaction between the subordinate and the supervisor. Therefore, supervisors should provide their support and direct supervision to subordinates in completing their tasks on daily-basis and meeting deadlines of projects. Further, in order to increase productivity, supervisors must urge their staff to set up a dedicated workstation at home. In this situation, the manager or direct supervisor plays a crucial role. Therefore, researchers recommend organizations focus more on that aspect.

Further, since individual workspace too with a p-value of 0.000 was found to have a significant impact, employees are encouraged to focus more on having a separate working area within the house premises to concentrate well on work. While at work, employees have separate workstations with individual equipment to assist their work so that their daily tasks can be completed without any interruptions. The same scenario should be applied when WFH by having an individual workspace to avoid unnecessary disturbances. In addition, companies might give employees who are experiencing high levels of work-life conflict more telework options. This might allay anxieties they have about juggling work and family obligations. The implementation of the WFH policy may provide numerous hardships for the staff, including poor coordination and ineffective communication. To build a productive remote working group that can communicate everyday via Google Meet, Zoom, Google Hangouts, and Skype, managers must establish good communication norms. Access to advantages like training opportunities and professional development activities may be restricted for remote workers. To make up for the loss of informal learning, companies might finance the formation and upkeep of a larger support network. They must also recognize the strain that comes with working remotely, pay attention to their worries and concerns, and show empathy for their struggles, especially when the change to remote work was sudden. Therefore, it is visible that from the variables considered in the study, supervisor’s trust and support and individual workspace significantly affect WFH.

Many past studies concentrated on circumstances in which WFH was typically a short-term choice for selected employees under unique circumstances. Now, with the impact of Covid-19, WFH may be a long-term alternative for most employees. Therefore, the results and recommendations of this study can be applied to other sectors as well in order to overcome the imbalance of work and life created due to WFH. This article expands the scant body of knowledge on the effects of WFH on Sri Lankan software engineers’ work-life balance following the pandemic, and future research into this topic should focus on other un-addressed factors such as employee’s mental wellbeing, productivity, family satisfaction that can affect the work-life balance of software engineers. These results hold true for every IT company, according to earlier studies. By taking care of these elements, firms may make sure employees are as productive in WFH as they are in the office. On the other hand, employees can focus more on the factors that would improve their work-life balance.

## 7. Limitations and directions for future research

The study was constrained to a variety of factors. Mainly this study used cross sectional data but longitudinal data would be better to identify the WFH’s pre-pandemic and post pandemic impact on work-life balance. Moreover, future research could analyze the impact of variety of industries rather than considering one industry, since the study focused only on IT and software development sector. For study purposes, the Western province was chosen as the geographical context, future researchers could expand the geographical context. Finally, since this study does not examine the qualitative approach, the validity of the findings is based solely on how well we can interpret and comprehend the current data set and explore key challenges.

## Supporting information

S1 Appendix Comparative table of previous literature. (DOCX)

S2 Appendix Framework of study design. (TIF)

S3 Appendix Tabular analysis of past literature. (DOCX)
