# Supplementary material for: Towards work-life balance or away? The impact of work from home factors on work-life balance among software engineers during Covid-19 pandemic
Source: PLoS One. 2022 Dec 14;17(12):e0277931. doi: 10.1371/journal.pone.0277931 (PMC9750026; doi:10.1371/journal.pone.0277931)
Supplement: S1 Appendix — (DOCX) [file pone.0277931.s001.docx]

**S1 Appendix. Comparative table of previous literature**

| 1. **Working conditions** | | | |
| --- | --- | --- | --- |
| **Author** | **Country** | **Findings** | **The gaps addressed in this study** |
| Kawachi (2008) | USA | A worker's health could be harmed by nonstandard work. | - Researchers have addressed the effects of poor working conditions on workers in a global scenario. Hence, this study addresses the Sri Lankan aspect and the software engineers specifically. - Other studies addressed this factor generally, while this study discusses this factor with regard to working from home during the Covid-19 pandemic. - Other studies have used different analyzing tools, while this study uses Structural Equation Modeling to analyze the data |
| Aleksynska et al. (2019) | 41 countries across the globe | The three primary metrics for analyzing working conditions are compensation, working hours, and contractual agreements. Working conditions must be quantified in order to be understood. |  |
| Strazdins et al. (2015) | Australia | Results highlight the social distribution, dimension, and health-related possibilities of time. |  |
| Bannai and Tamakoshi (2013) | Japan | Working long hours is associated with depressive state, anxiety, sleep condition, and coronary heart disease |  |
| Lunau et al. (2014) | 27 European countries | Employees who complained about a poor work-life balance experienced more health issues. |  |
| Lenhart (2016) | 24 Organization for Economic Co-operation and Development (OECD) countries | Higher minimum wage levels are linked to significantly lower overall death rates. |  |
| Bouncken, Lapidus and Qui (2022) | No specific country | A better work-life balance, more autonomy and flexibility, and more productivity are all benefits of working from home. |  |
| 1. **Supervisor’s trust and support** | | | |
| **Author** | **Country** | **Findings** | **The gaps addressed in this study** |
| Maertz et al. (2007) | USA | Low perceived supervisor support strengthened the negative relationship between  Perceived organizational  Support and turnover, while high perceived supervisor support weakened it. | - Other studies have not addressed a specific workforce, while this study investigates the effect of a supervisor’s trust and support factor on software engineers’ perspective |
| Den Dulk and de Ruijter (2008) | UK and the Netherlands | Supervisory requests are not as well received as ones from other parties. |  |
| Ru Hsu (2011) | Taiwan | Perceived supervisor support and internal locus of control significantly mitigate the link between work-family conflict and job satisfaction. |  |
| Den Dulk et al. (2016) | Netherlands and Slovenia | The quality of the leader-member exchange relationship, general support, and the importance of specialized family support varies depending on the environment and the results considered. |  |
| Skiba and Wildman (2018) | USA | Organizations interested in enhancing trust between supervisors and subordinates would be good to assess and manage both employee and supervisory trust. |  |
| Abendroth and Dulk (2011) | 8 European countries | Organizations interested in enhancing trust between supervisors and subordinates would be good to assess and manage both employee and supervisory trust. |  |
| Bailyn (2011) | USA | Work can be rearranged to support employees' work-life balance and the efficiency of the business. |  |
| 1. **Possibility to access organization’s networks at home** | | | |
| **Author** | **Country** | **Findings** | **The gaps addressed in this study** |
| Golden (2021) | USA | The greatest teleworkers pay close attention and practice discipline in order to effectively manage the work-home divide. | - These studies addressed their different objectives by considering this factor while researchers conducted this study in order to address the relevant factor regarding working from home during the Covid-19 pandemic. - In this study researchers tend to cover up the gap which is whether this factor has a significant impact on work-life balance. - Researchers tend to demonstrate this factor in the Sri Lankan context in software engineers’ perspective since no past study has been found related to it. |
| Anderson and Kelliher (2020) | UK | Women, particularly mothers, have had little option in terms of when and how much labor they undertake. |  |
| Islam (2022) | India | To comprehend emerging work experiences around the world, the picture of a well-paid white-collar worker (usually a woman) working from home is no longer adequate. |  |
| Sellar and Peiris (2021) | Sri Lanka | Findings resulted that specifically on women, there's a significant impact of WFH on job satisfaction |  |
| Cooper and Kurland (2002) | USA | Employees in the public sector are less likely than those in the private sector to be hindered by telecommuting in terms of their professional growth. |  |
| Pashchenko (2021) | Different countries from Russia to USA | A new trend in software development, hiring specialists, and team organization that is related to high-tech IT businesses' resistance to resume collaborating in shared offices must be promptly understood. |  |
| 1. **Number of children** | | | |
| **Author** | **Country** | **Findings** | **The gaps addressed in this study** |
| Zhang et al. (2020) | Germany | It was found out the factor of having or not having children is the main effect which determines whether the individual likes to WFH or not. | - These past studies have not addressed this factor by considering Sri Lankan context with regard to software engineers. Therefore, it led researchers to cover up the research gap through this study. |
| Rathnaweera and Jayathilaka (2021) | Sri Lanka | It was proven that the work-life balance of employees is significantly affected by both working and non-working environments, further the non-working environment's impact was larger on this relationship. |  |
| Ajjan et al. (2020) | 38 countries including America & Germany | Results that workers' control over time, technology usefulness, and WFH conflict are influenced by gender and the presence of children under the age of 18, but not by their WFH attitude. |  |
| Carli (2020) | Asia, Africa, North and South  America and Oceania | Telecommuting has the potential to expand men's family duties in the long run, lowering the gender gap in home chores and boosting gender equality. |  |
| Semlali and Hassi (2016) | Morocco | Researchers found that strategies such as increasing the duration of maternity leave, having nurseries at work, and working from home could assist women in achieving work-life balance. |  |
| Crosbie and Moore (2004) | Ireland | Study emphasis on work-life balance should be appreciated since it may allow for more flexibility in the workplace for those organizations who take it seriously.  Providing options for a wide variety of workers. |  |
| Panisoara and Serban, (2013) | Romania | The results demonstrate that the levels of work-life balance across the four employee types studied—unmarried, married without children, married with children under 18, married with children over 18—are not significantly different. |  |
| 1. **Individual workspace** | | | |
| **Author** | **Country** | **Findings** | **The gaps addressed in this study** |
| Tokdemir (2022) | Turkey | The dependent variables are thought to have a positive relationship with resource-related protective factors and a negative relationship with job strain. | - Even though past studies elaborate this factor in order to address different scenarios, no study has been found which had discussed about this factor with regard to IT sector in Sri Lankan context during Covid-19. Therefore, researchers observed the research gap and tend to elaborate it through this study. |
| Shirmohammadi, Au and Beigi (2022) | 21 countries including United States, India, Germany, | Children are important in the behavior of teleworkers. It not only exacerbates conflict between work and family, but it also causes couples to divide up the chore of housework. |  |

Source: Authors’ compilation based on past literature
