# Supplementary figures and images for: Towards work-life balance or away? The impact of work from home factors on work-life balance among software engineers during Covid-19 pandemic
Source: PLoS One. 2022 Dec 14;17(12):e0277931. doi: 10.1371/journal.pone.0277931 (PMC9750026; doi:10.1371/journal.pone.0277931)

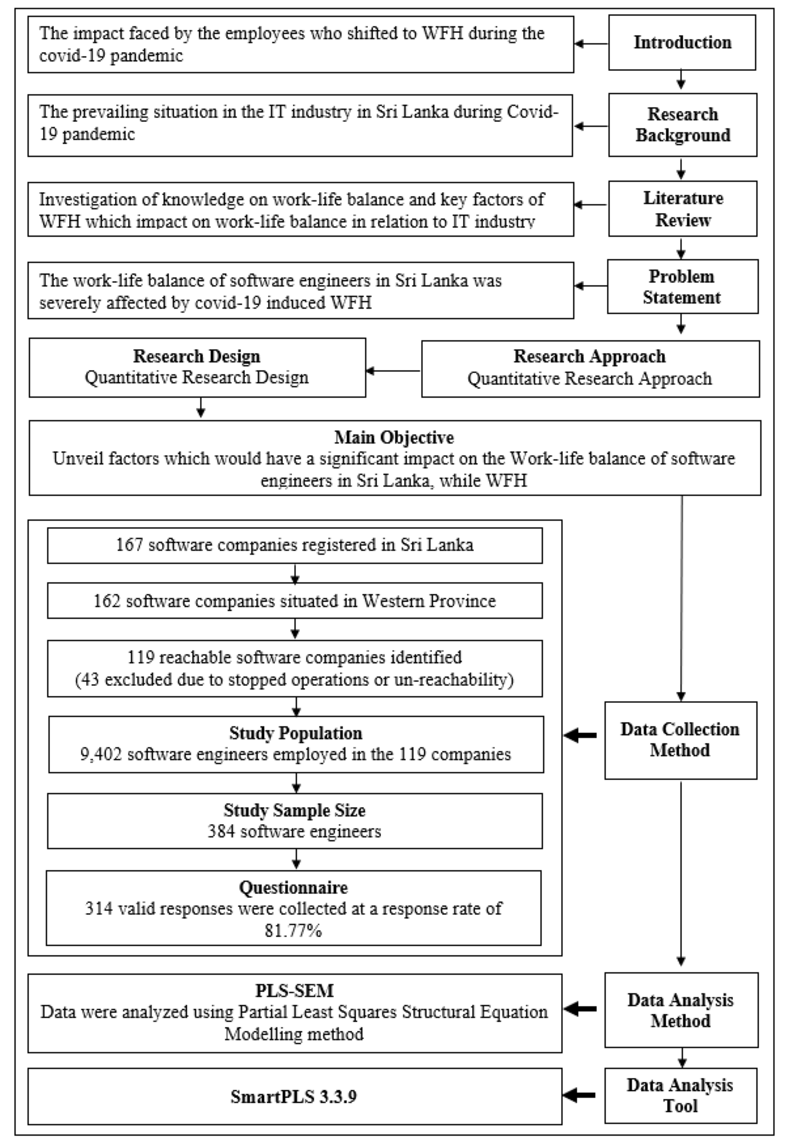

Supplement: S2 Appendix — (TIF) [file pone.0277931.s002.tif]
