# Supplementary material for: Towards work-life balance or away? The impact of work from home factors on work-life balance among software engineers during Covid-19 pandemic
Source: PLoS One. 2022 Dec 14;17(12):e0277931. doi: 10.1371/journal.pone.0277931 (PMC9750026; doi:10.1371/journal.pone.0277931)
Supplement: S3 Appendix — (DOCX) [file pone.0277931.s003.docx]

**S3 Appendix. Tabular analysis of past literature**

| **Objective** | **Hypothesis** | **Literature** | **Methodology** | **Results/ Findings** |
| --- | --- | --- | --- | --- |
| To examine the factors that have a significant impact on work-life balance of software engineers. | H_1_(WC) | Wolor, Nurkhin and Citriadin (2021) | Structural Equation Modeling | Having the right conditions while WFH does have a significant impact on work-life balance. |
|  |  | Gibbs, Mengel and Siemroth (2021) | Regression Analysis | Working hours and time spent on meetings have been increased during WFH, hence has resulted in a decrease of productivity, and is anticipated that this might impact on work-life balance. |
|  |  | Bannai and Tamakoshi (2013) | Systematic search for articles between 1995–2012 | Working hours have a significant impact on work-life balance. |
|  |  | Islam (2022) | Exploratory framework | It is required to have proper equipment and connectivity when employees WFH. |
|  | H_2_ (STS) | Abendroth and Dulk (2011) |  | Supervisor’s support does impact on work-life balance. |
|  |  | Den Dulk et al. (2016) | exploratory factor analysis/multiple regression analyses | Supervisory support helps to achieve an increment in work-life balance. |
|  | H_3_(PAON) | Sellar and Peiris (2021) | Descriptive Analysis/ Multiple Linear Regression | Possibility to access organizations networks does not significantly affect to job satisfaction. |
|  | H_4_(NOC) | Panisoara and Serban (2013) | Descriptive Analysis using SPSS | The presence of children has no significant impact on the work-life balance. |
|  |  | Ajjan et al. (2020) | Two-way Multivariate Analysis of Variance (MANOVA) | Having children significantly impacts on work-life balance. |
|  |  | Rathnaweera and Jayathilaka (2021) | Regression analysis | Having children significantly impacts on work-life balance. |
|  | H_5_(IW) | Golden (2021) | Exploratory framework | A separate workspace leads to a better boundary management between work and home. |
|  |  | Shirmohammadi, Au and Beigi (2022) | Thematic analysis | Designated workspace is of vital need to achieve work-life balance. |

Source: Authors’ compilation based on past literature.
